# Human Motion Recognition of Knitted Flexible Sensor in Walking Cycle

**DOI:** 10.3390/s20010035

**Published:** 2019-12-19

**Authors:** Yutian Li, Xuhong Miao, Li Niu, Gaoming Jiang, Pibo Ma

**Affiliations:** Engineering Research Center for Knitting Technology, Ministry of Education, Jiangnan University, Wuxi 214122, China; 7170707015@stu.jiangnan.edu.cn (Y.L.); 7180707007@stu.jiangnan.edu.cn (L.N.); jgm@jiangnan.edu.cn (G.J.); mapibo@jiangnan.edu.cn (P.M.)

**Keywords:** knitted fabric, large strain sensor, flexible sensor, motion recognition, gait cycle

## Abstract

Knitted fabric sensors have been widely used as strain sensors in the sports health field and its large strain performance and structure are suitable for human body movements. When a knitted structure is worn, different human body movements are reflected through the large strain deformation of fabric structure and consequently change the electrical signal. Here, the mechanical and electrical properties of highly elastic knitted sweatpants were tested under large strain. This sensor has good sensitivity and stability during movement. Compared with traditional motion monitoring, this technique divides the walking cycle into two stages, namely, stance and swing phases, which can be further subdivided into six stages. The corresponding resistance characteristic values can accurately distinguish the gait cycle. Analysis on hysteresis and repeatability revealed that the sensor exhibits a constant electrical performance. Four kinds of motion postures were predicted and judged by comparing the resistance characteristic range value, peak value calculation function and time axis. The measured sensor outputs were transferred to a computer via 4.0 Bluetooth. Matlab language was used to detect the status through a rule-based algorithm and the sensor outputs.

## 1. Introduction

Flexible conductive materials can potentially be applied in human motion sensors, health monitoring for medical monitoring systems such as respiratory rate, heart rate and body posture to human–machine interface and wearable integrated devices that have attracted widespread attention [1,2,3,4,5,6,7,8,9,10]. Most common flexible sensors are made of conductive metal nanoparticles, metal films, carbon nanotubes and graphene [11,12]. Every material has its own conductivity; however, their working strain range is small and thus limits their practical application [8,13,14]. In the design of a strain sensor corresponding to human movement, the following key factors must be considered—large strain range, fast recovery deformation and high sensitivity [15,16,17]. Some special sensors (such as electromyographic signal) can be used to sense the activity of muscle and have great potential for human movement detection [5,18,19]. However, most of these systems require additional hardware that increases the costs, reduces user comfort and are not easily integrated into other wearable devices [20]. Other researchers considered motion sensors, such as smart bracelets and watches that use multi-axis acceleration sensors and gyroscope sensors to calculate steps and distance recognition; however, these sensors cannot accurately identify human movements [10,21]. In addition, algorithm problems induce errors in calculating steps, thus making the results greatly different from the actual situation [22,23]. Strain sensors are one of the key components of the interface between human motion and electrical signals. Some highly sensitive strain sensors made of nanomaterial and graphene materials can detect small movements, such as vocal cord vibration detection, pulse detection and respiratory monitoring [15,19,24]. Conductive fabrics are a promising material due to their high flexibility, comfort to human skin and ease of weaving and integrability with other wearable sensors [25,26]. Conductive properties are introduced into text-based strain sensor by integrating flexible devices into the fabric, embedding conductive yarns into the fabric structure or coating the fabric surface with conductive materials; the textile strain sensor can be formed using yarn or fabric and through the electrochemical method [26,27,28]. The circuit path is formed through internal interaction and is induced by the large or small deformation of fabric caused by human movements. Given the high elasticity of coil structure, textile is one of the most common materials for a sensor structure and knitted conductive fabric is suitable for human movement and stretch recovery and thus can be used as flexible strain sensor [29,30]. 

The knitted flexible sensor exhibits the characteristics of high-elastic sports tights, which are directly in contact with the human skin and restores the stretched skin during movement, human comfort and sensor signal accuracy [31,32]. This material can also meet the characteristics of large strain and repeatability during human movement and accurately represent walking through electrical signals [33,34,35]. 

This paper reports a sensor made of highly elastic knitted sweatpants with high sensitivity and stability during large-strain movement such as walking. Differences in the of characteristic values of resistance in each stage were analyzed by subdividing stance and swing phase into six stages, the hysteresis and repeatability of large-strain knit flexible sensor were verified and the range of characteristic value data was established for each subdivided motion mode. Finally, peak function and time axis were compared to determine whether the knitted flexible sensor can identify large-strain human motion.

## 2. Materials and Methods 

**Figure 1:**
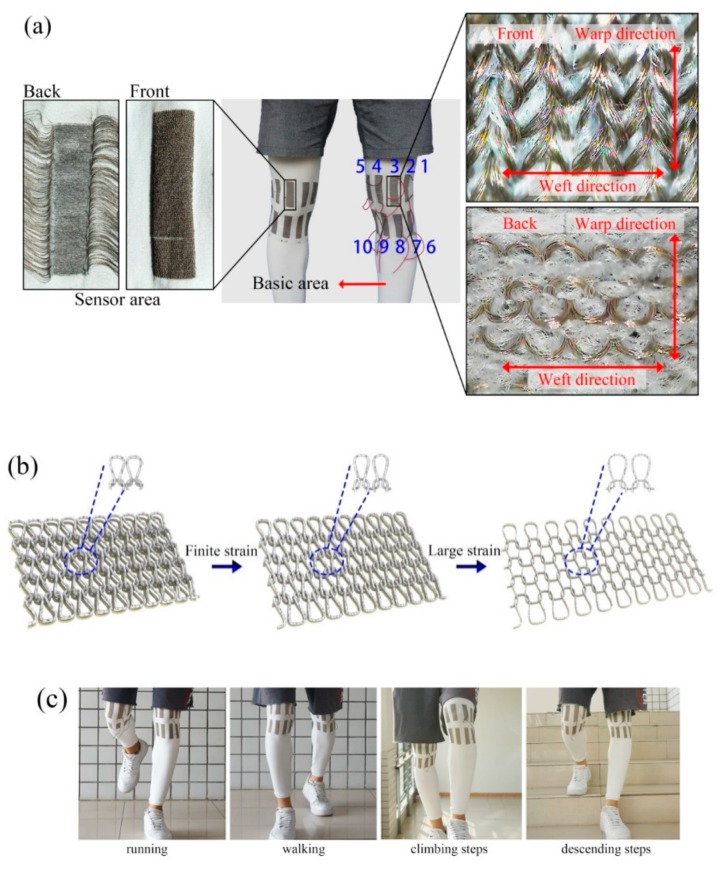
(**a**) High elastic knit strain sensing stretch pants with ten sensing areas. (**b**) Deformation of knitting loops (**c**) The subjects completed four different activities such as running, walking, climbing and descending steps.

The knitted sweatpants were knitted by plated stitch to prepare highly elastic sports pants with sensing performance. Figure 1a shows a sample of the highly elastic knitted sweatpants with 10 conductive strain sensors in the leg region and a conductive sensing area around the knee joint that can meet most of the knee joint movements. The white area is 40D polyamide and polyamide wrapped spandex and the sensing area is 40D silver-plated polyamide and polyamide wrapped spandex. The silver-plated yarns in the sensing area were knitted by float stitch and each sensing area has the following dimensions—5 cm long and 1.5 cm wide. In addition, the fabric’s off-loom density is P_A_ × P_B_: 25 wales/cm × 17 courses/cm. The materials were obtained from Hengtong X-Silver Speciality Textile Inc. On the SM8-TOP2 MP2 (0.907 mm, E28, 15 inch, Santoni Spa, Italy). The area around the knee has the most remarkably deformation of the fabric; hence, the size of the sensor for this part (5 cm long and 1.5 cm wide) is appropriate. Previous research on weft knitting conductive fabrics reported that the largest area of knee joint movement variation can be covered [36,37]. The 10 sensors were designed to determine what part of the sensor around the knee accurately represents a walking person’s gait. Non-conductive polyester and conductive yarns were mixed and knitted to stabilize the structure between the sensor section and the base of the sweatpants. The sensor number is 1–10, hereafter referred to as S1–S10.

The resistance of leg movement was monitored in real time with a Rigol multimeter (DM3058 digital multimeter) to evaluate the resistance variation characteristics. A digital multimeter was used to separately test the resistance signals in ten areas. In our previous test, we used our own resistance acquisition software with Bluetooth 4.0 protocol to transmit and communicate. This software can transmit 20 resistance values per second and convert the electric signal into digital signal by A/D conversion circuit, external pair. The specific resistance was 100 Ω and the resistance value of the sensor was calculated using the properties of the series circuit [38]. In general, elastic knitted fabrics have tight contact points between their loops. In pre-stretch fabrics, the loops are squeezed tightly together due to the compact effect of highly elastic knitted pants. After stretching, the distance between the loops increases as shown in Figure 1b. The adjacent circuits formed by the conductive silver-plated yarn in the sensing area come in contact with each other and generate electrical conductivity. The highly elastic surface provides the ability to match the shape of the user’s legs, thereby improving comfort and fitness. Therefore, the reduction of surface contact points and the deformation of the loop due to stretching when matched with the user may increase the initial resistance compared with that under the restored state. Given the high elasticity and recovery effect of highly elastic knitted structures, the hysteresis of the resistance behavior under large strain tensile condition must be studied.

Two contact electrodes were connected to the specified stretch and recovery area to measure the resistance on the knitted fabric surface and resistance variations were studied. The floating line was hooked on the opposite side of the fabric to the front and welded together with the two electrodes by using the welding material as the metallic tin. The relationship between resistance and time of knee joint in running, walking, climbing and descending steps was tested. Ten subjects (five males and five females, age range of 20–30 years) performed four different activities according to a fixed sequence and experimental protocol shown in Figure 1c. A large data set must be collected to meet the identification needs suitable for many users. Thus, the subjects were asked to perform each activity for approximately 2 min.

For lag performance, the fabric was observed by the sensor resistance within the process of stretching-reply to prevent the conductive fabric from stretching at the same rate (100 mm/min) and to change the maximum stretching of the fabric. The same fabric was stretched by 15%–50% and the resistance change was captured during fabric stretching recovery in real time. For cyclic performance, the leg repeated motion was captured in real time over 500 times. One area was selected for 10 min of repetitive walking and 60 cycles of walking per minute were recorded. Resistance change was also observed. 

## 3. Results and Discussion

### 3.1. Suitable Areas for Sensing

**Figure 2:**
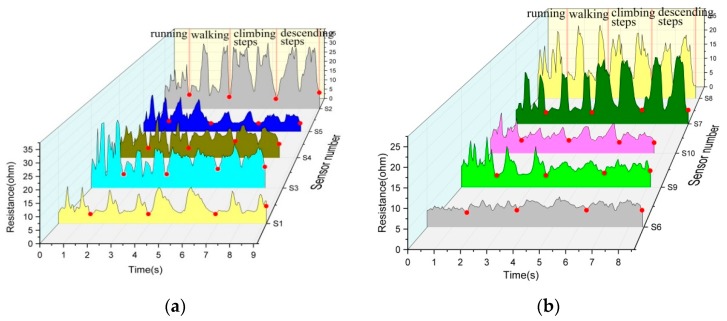

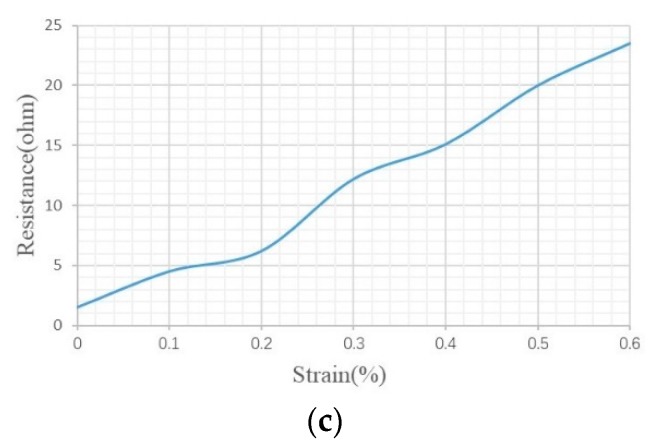
(**a**) and (**b**) Resistance changes of knee joint continuous motion in four states: running, walking, climbing and descending steps. (**c**) Knitted sensor calibration curve.

Figure 2a shows that the resistance changes in each cycle are quite similar but still have their own characteristics, in which each red dot represents the end of an action. Thus, the highly elastic conductive knitted sweatpants can accurately reflect the change of resistance and exhibits high sensitivity. The sensitivity of strain sensors is usually expressed by the evaluation index factor GF. According to the review by Xie and Zhang, the GF value of most strain sensors ranges from 0.42 to 5; hence, the sensitivity of knitted sensors herein is 2.73 [9,39]. The relative resistance of 100 Ω was used as a voltage divider and connected in parallel with the sensor. The resistance value of the sensor was measured with a digital multimeter to accurately reflect the resistance change.

$$\mathrm{GF}=\frac{\Delta R/R_{0}}{\Delta L/L_{0}}$$

According to the varying resistance waveforms of the 10 regions, those of S1, S4, S7 and S8 are relatively similar. The number of peak and valleys vary similarly in this range of amplitude. In the whole database, the change of each peak valley corresponds to the walking movement of knee joint. Under the four motion states, the variation of resistance is similar when running and walking or when going up and down the steps. This finding is not conducive for identifying the motion pattern as a sensor. In S2 and S6, the resistance is lower than that in the other three sports states and the change of resistance is small. The change of electrical resistance is relatively clear when walking and is similar when moving up and down the steps. Hence, this area can be used to judge the running movement.

In S3, S5, S9 and S10 the changes of resistance waveform in each region are different and the resistance is higher during running than during the other three movements. When walking, the change of resistance is clear. A big difference is observed between the upper and lower steps in S3 and S10, whereas the change of resistance is similar in S5 and S9. Although fabric sensors have shown excellent performance in human motion recognition and other fields, most sensors are single or integrated as additive parts in the test [5,40]. In this work, conductive yarns were directly positioned and knitted into elastic leggings to test different area sensors and determine the best sensor area.

S3, which is located in the middle of the knee joint and has relatively high strain tensile property can accurately reflect the human gait cycle. The range of movements and resistance changes are relatively high when running. The four kinds of movements in the walking cycle are consistent with the resistance changes. The characteristics of resistance waveform conform to the law of movement change, which is suitable for use as a large strain sensor. S10 is not suitable as a sensor because it is located in the inner part of the leg. Its stretching recovery is not stable and the resistance characteristics of walking and descending steps have similar points. In summary, S3 is located in the mid-knee region with large knee strain variation and thus has the clearest change of resistance waveform in each motion cycle. The calibration curve of the sensor in S3 region was measured and a linear relationship was observed between the resistance and strain as shown in Figure 2b.

### 3.2. Resistance Waveform Corresponds to the Gait Cycle

According to the kinematic characteristics of the human body, human walking movement can be divided into stance period and swing period and can be further subdivided into six stages [41]. The stand phase is the time from one side of the foot to the same side of the toe and the swing phase is the time from the tip of the toe to the heel on the same side. The line segment changes corresponding to each action are subdivided into (a) early stance, (b) mid stance and (c) late stance; (d) pre-swing, (e) mid-swing and (f) terminal swing phase.

**Figure 3:**
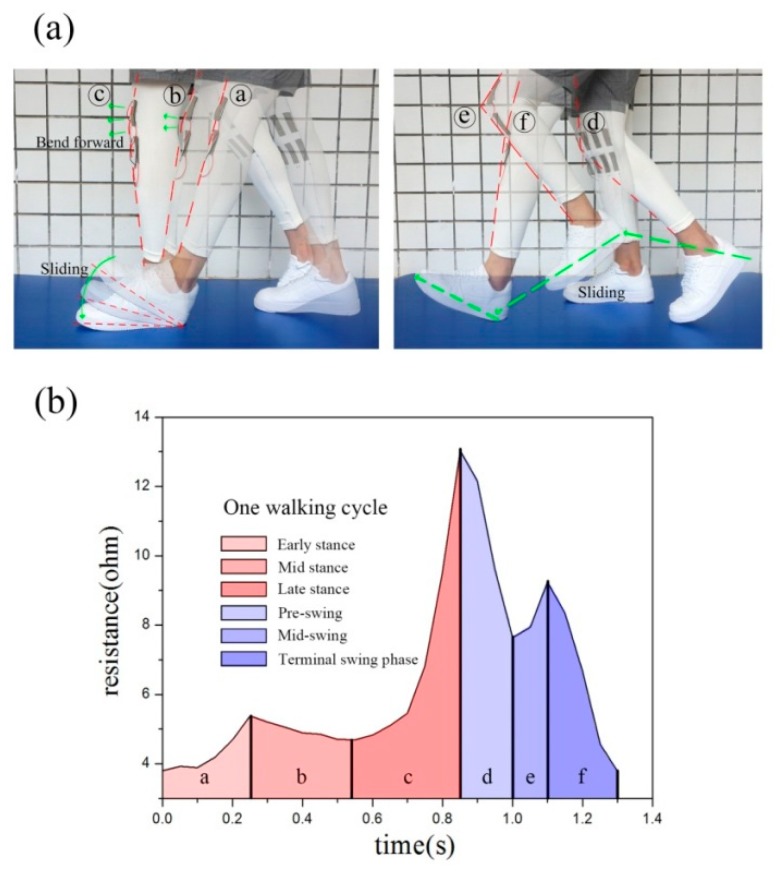
(**a**) A complete walking cycle of six stages; (**b**) Variation of walking cycle resistance in S3.

As shown in Figure 3a, the resistance change of each cycle clearly exhibits stance and swinging periods. 

The resistance was analyzed according to the walking cycle of S3 selected above as shown in Figure 3b. Red areas a–c correspond to the early, mid and late stance, respectively, in each subdivided movement mode under walking cycle. The blue areas d–f correspond to the pre-swing, mid-swing and terminal swing phase, respectively. In the early stance, area a is heeled to full sole, the knee joint is slightly bent and thus the resistance increases slightly. In the mid stance, area b is full soles landing to heel off the ground, the knee joints slowly straighten and thus the resistance decreases slightly. In the late stance, area c is from heel to toe of the ground, the knee joint significantly bends forward, the body starts to move forward and the resistance increases significantly.

In the pre-swing, the area d is the accelerated forward swinging of the legs and the forward swinging of the knees. Joint angles are straightened rapidly, the body moves forward and the resistance decreases rapidly. In the mid-swing, zone e is the resistance hysteresis reaction that occurs when the maximum tensile value of the fabric is reached during the forward swing of the leg. Hence, the resistance increases at this time. In the terminal swing phase, the area f gradually straightens all the legs and knees and the resistance decreases rapidly until the minimum value is reached. At this time, the position of the heel touching the bottom is ready to enter the early stage of support again.

**Figure 4:**
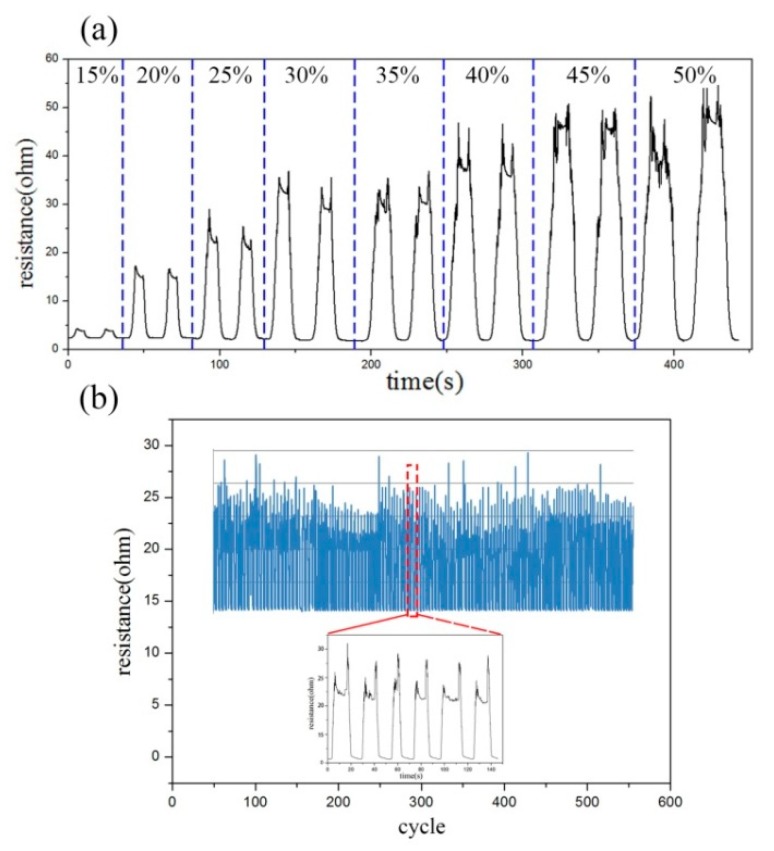
(**a**) Hysteresis of fabric stretch-recovery under large strain; (**b**) Variation of resistance and circulation under 500 cyclic tensile strains.

The conductive fabric resistance shows hysteresis after the cyclic stretching of large strain, due to the large strain effect. This finding verifies that the knitting sensor area enters the mid-swing. With the same stretching rate, the same sensing area was stretched by 15%–50%. Figure 4a shows that at a low strain variable of 15%, the resistance of the sensing area shows good monotonicity, increases during stretching and decreases during recovery. However, after the tensile amount reaches 15% and 20%, a second increase is observed in the resistance curve. Thus, when the fabric is stretched, the resistance increases slightly and then decreases to form the first peak. The same phenomenon occurs in the stretch-recovery to form the second peak.

This phenomenon is called the hysteresis of fabric stretch-recovery. The fabric has a low tensile recovery rate and the strain recovery is relatively slow after a large stretch. At this time, the hysteresis of fabric tensile recovery shown on the tension/strain curve becomes highly evident when the tensile amount exceeds 30%. The hysteresis is caused by the fabric structure, the friction of yarn and the friction between fibers.

The durability of knitted stretch pants sensor system is another key characteristic of the sensor system and is reflected in the cycling performance. In our daily stability test, the test was conducted either for over 30 min or to a distance of 3 km. The electrical signal produced by a human while walking and the change of electrical resistance signal during 500 cycles were recorded. The continuous cycle shows similar and stable law in the test process, indicating that the sensor has good stability in the long-term cycle of deformation. Figure 4b shows that most studies only distinguished the changes of resistance waveform during human movement; hence, identification is not possible [16,42]. In this study, each walking posture was subdivided into six periods and the movements within the six periods corresponded to the resistance waveform point by point.

### 3.3. Resistance Characteristic Value and Recognition of Human Gait Cycle

**Figure 5:**
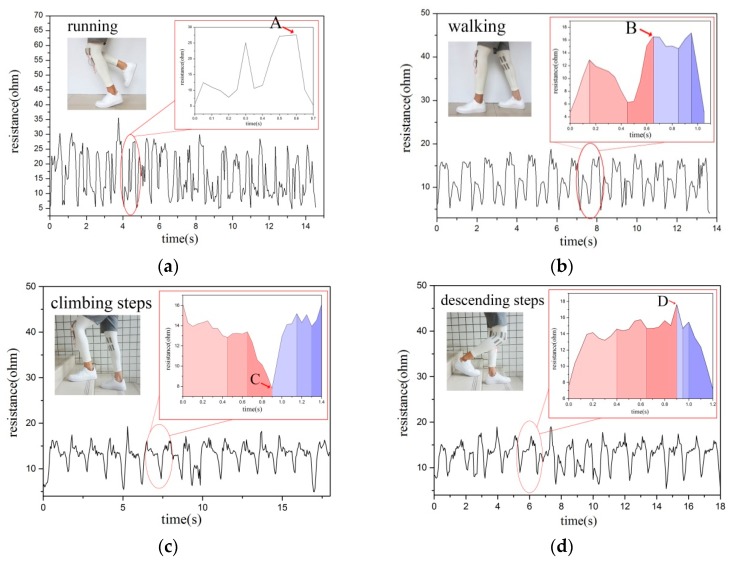
Relationship between resistance and time within the six subdivided stages under four human motion states. (**a**) Resistance and characteristics when running; (**b**) Resistance and characteristics when walking; (**c**) Resistance and characteristics when climbing steps; (**d**) Resistance and characteristics when descending steps.

According to the above various sports modes, the knee joint angles in each sport cycle have their own characteristics and certain commonalities even during running, walking, climbing steps, descending steps or sitting up and down. Through observation, we can see that the variation range of resistance in running cycle is significantly larger than that in the other three sports modes. During running, the range of motion is relatively large, the resistance changes rapidly and the maximum resistance is larger than that of the other three actions. Therefore, the maximum resistance is used to identify the running state. In running, the leg exhibits significant backward and upward movements, thus establishing a maximum value point A of the resistance at this time. The maximum range is 25–30 Ω as is shown in Figure 5a.

According to the above analysis of the six stages of the walking cycle, the maximum of knee joint resistance point B is observed between the stance and swing period, as is shown in Figure 5b.

The resistance change of the knee joint during ascending steps is opposite to that under the other three motion states. This phenomenon occurs because the leg is in a raised state during the swing phase of the upper step and the knee joint angle is large at this time. In the movement climbing steps, the angle begins to decrease gradually during the supporting period, leading to the resistance rapidly reaching a maximum value in the upper step cycle. Upon entering the stand phase, the angle gradually decreases until the resistance minimum point C is achieved in the late stance, as is shown in Figure 5c. The change of knee resistance in the lower step is completely different from that in the upper step. At the early stance, the knee angle is at the minimum value but it gradually increases. After a short period of time from the late stance to the pre-swing, the knee angle reaches the maximum value. Therefore, the sensor resistance reaches the maximum value D, as is shown in Figure 5d.

The peak and trough points were used to accurately judge the six stages. First, in walking, climbing and descending steps, the maximum and minimum values of a period were applied to distinguish the stance and swing phases. Second, the maximum, minimum and sub-maximum values of the stance and swing phases were subdivided into the anterior, middle and posterior periods.

The characteristic value of the electrical resistance signal of the knee joint angle was extracted by using the mean electrical resistance ratio, which refers to the average resistance during the stance and swing phase of walking, climbing and descending steps. The average value of knee resistance in each subdivision mode of motion was calculated successively in the early, mid and late stances and the pre-swing, mid-swing and terminal swing phases. Finally, the ratio of the average knee resistance of each movement subdivision mode to the average knee resistance during the stance or swinging phase was used as the characteristic value T of knee angle in this movement mode.

R1=∑i=1niRini,R=∑i=1NiRiNi,T=R1R,

where R_i_ is the specific knee resistance value at a certain moment within the sampling interval. N_i_ is the total number of sampling points in the corresponding period (stance and swing phase). R is the resistance during the stance or swing period. n_i_ represents the total number of sampling points under specific subdivision modes (early stance, mid stance and late stance; pre-swing, mid-swing and terminal swing phase). R_1_ is the resistance values of each six subdivisions. Table 1 is the resistance eigenvalue range for the six stages of each subdivision mode calculated from 500 gait cycles and the range was obtained from T values using the above formula. The characteristic value information of resistance in different motion modes has different characteristics and good discrimination.

Table 2 shows three dynamic features extracted from the sensing S3 in a certain period. First, the motion stance and swing phase were determined by resistance waveform and the six subdivision stages were divided to calculate the characteristic value of resistance in six stages (Table 2). Moreover, the corresponding motion posture can be judged by comparing the obtained eigenvalue range with the eigen database. The results showed that the some data do not conform to the eigenvalue range. In addition, the prediction matching degree of resistance eigenvalue is 62.5%, which can be used to judge the four dynamic characteristics of the lower limbs of the human body.

Through the above method of resistance characteristic value, we can predict the state of human movement in a certain period of time. However, the prediction accuracy of 62.5% needs to be improved. Therefore, we used the following two methods to conduct the second and multiple mutual verification of judgments for the accurate identification of human movement.

**Figure 6:**
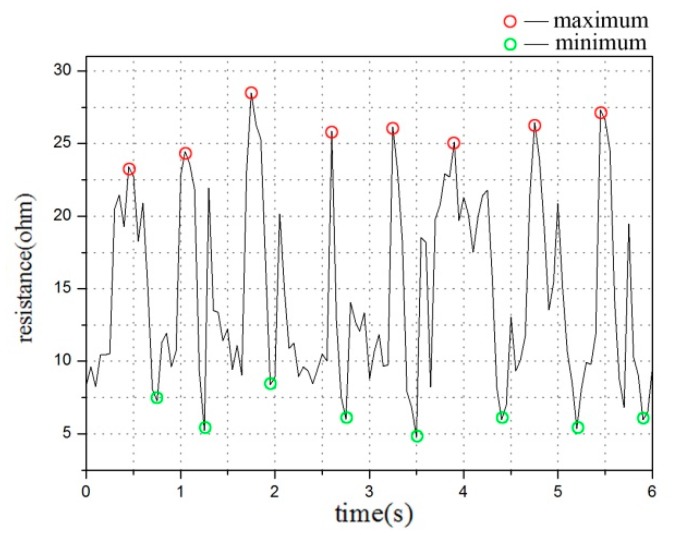
Peak computing function of Matlab used to findpeaks and troughs within unit time.

First, we used the peak computing function findpeaks of Matlab to seek peaks and valleys within unit time and conduct data detection. The movement characteristics and our resistance waveform show that, in unit time, the highest resistance change period and the maximum and minimum values of the resistance are observed when the human body is running. These values are all relatively larger than those of the other three movement periods. With the use of this algorithm, we accurately distinguished running from the other three sports (Figure 6).

In the other three movement stages, during the movement of the climbing and descending steps, resistance rises or falls singly in the stance and swing phases. In addition, when the step moves up, the resistance rises in the swing phase and decreases in the stance phase. On the contrary, when the step moves down, the resistance falls in the swing phase and rises in the stance phase. Different from the other two movements, going up and down the steps in the stance phase requires a longer time compared with in the swing phase.

**Figure 7:**
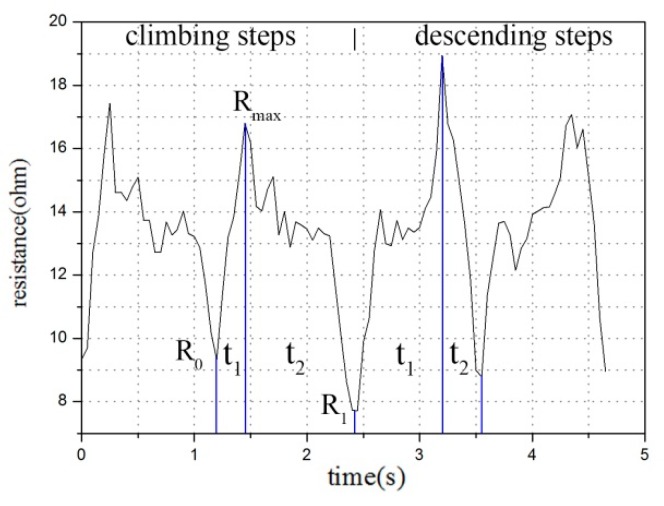
Time-axis comparison analysis of up-down step motion.

Comparison using the method of time axis comparison states that the movement is a climbing or descending step, when only one maximum value of resistance is observed within the same period and the change of resistance value is monotonous (Figure 7). If the time (t_1_) taken from the initial resistance (R_0_) to the maximum resistance (R_max_) is greater than the time (t_2_) taken from the end resistance (R_1_), then the movement is descending steps. By contrast, if the time t_1_ is less than t_2_, then the movement is the climbing steps. Extracting the characteristic value by fitting the linear function or matrix and using calculus is relatively complicated and difficult. [9,11,15] In this work, the characteristic range value of the resistance, the calculation function of the peak value and the time comparison of the peak and valley point were used for evaluation.

### 3.4. System Operation

We do data processing by using Matlab language. The main processing steps are as follows.

**Figure 8:**
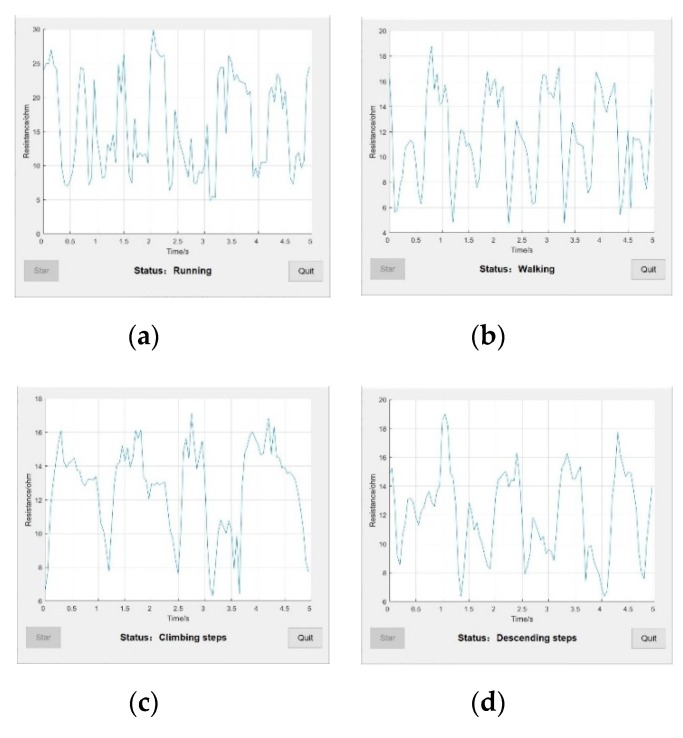
Snapshots of motion recognition display interface and output state corresponding to four motion states. (**a**) System status while running; (**b**) System status while walking; (**c**) System status while climbing steps; (**d**) System status while descending steps.

First, the collected data were validated and abnormal results were removed. A total of 100 data points at a time were examined to determine the starting point position of each cycle. By adding smoothing and derivative algorithm to the system, t_1_ and t_2_ were determined twice, which makes the accuracy of the whole system reach 78%. Within the same time period, the number of peaks and valleys and the amount of resistance amplitude, corresponding to the output running or walking state were analyzed. The start-end position and the peak point were then divided and the time relationships t_1_ and t_2_ were compared with output from the climbing step or descending step states as shown in Figure 8.

## 4. Conclusions

We studied the application of silver-plated conductive fiber to directly fabricate a flexible sensor on sports pants and achieve the long and comfortable sensing of leg movements in different gait states. The sensors placed at different positions have different sensing performances for motion. This finding is mainly related to the stretching and elongation of sensors at different positions caused by the motion of joints and the response of resistance to the recovery of deformation. The sensor located in the middle of the knee joint can reflect the difference of resistance change in different gaits. Under the four gaits of running, walking, climbing and descending steps, the change of sensor resistance signal highly correspond with the stance and swing period of human leg movement. According to the peak-valley detection method, walking, climbing and descending steps can be further subdivided into six stages.

The range of angle characteristic value of the knee joint was extracted by the mean resistance ratio. Four kinds of movements were preliminary judged by using the characteristic range value of resistance. In addition, four kinds of gait were recognized by extracting the peak value of resistance, valley value and time ratio of peak point in the gait period.

A gait motion recognition system is demonstrated, which can output the motion state according to the resistance waveform. The deficiency of this paper is the lack of mutual verification between two or more sensor areas and the test of motion diversity. For future work, we will use the data algorithm model to establish the database of motion input and output, simulate human body movement and establish an accurate identification.

## Figures and Tables

**Table 1:** Eigenvalue range of each subdivision mode.

	Walking	Climbing Steps	Descending Steps
Early stance	0.86 ± 0.3	1.08 ± 0.25	0.83 ± 0.3
Mid stance	0.91 ± 0.2	0.99 ± 0.2	1.01 ± 0.25
Late stance	2.01 ± 0.3	0.89 ± 0.45	1.10 ± 0.4
Pre-swing	1.24 ± 0.25	0.61 ± 0.4	1.25 ± 0.25
Mid-swing	1.35 ± 0.25	0.78 ± 0.2	0.90 ± 0.2
Terminal swing phase	0.83 ± 0.2	1.15 ± 0.3	1.31 ± 0.2

**Table 2:** Characteristic values of resistance in six stages at a certain moment in the sensing S3.

	Walking	Climbing Steps	Descending Steps
Early stance	0.95	1.43	0.63
Mid stance	0.88	0.74	1.42
Late stance	2.3	0.71	1
Pre-swing	0.85	0.78	1.7
Mid-swing	0.76	0.68	0.86
Terminal swing phase	0.97	0.77	1.66
